# Determinants of Immunoglobulin‐G N‐Glycan Patterns in the Aging General Population

**DOI:** 10.1111/all.70361

**Published:** 2026-04-30

**Authors:** Samantha Leonard, Ralph Heijmans, Virgil A. S. H. Dalm, Silvan Licher, Jan Nouta, Steinar Gijze, David Falck, P. Martin van Hagen, Robin Peeters, Manfred Wuhrer, Layal Chaker

**Affiliations:** ^1^ Department of Internal Medicine, Division of Allergy & Clinical Immunology Erasmus MC University Medical Center Rotterdam the Netherlands; ^2^ Department of Epidemiology Erasmus MC University Medical Center Rotterdam the Netherlands; ^3^ Department of Immunology Erasmus MC University Medical Center Rotterdam the Netherlands; ^4^ Department of Primary Care Erasmus MC University Medical Center Rotterdam the Netherlands; ^5^ Center for Proteomics and Metabolomics Leiden University Medical Center Leiden the Netherlands; ^6^ Center of Excellence for Allergy and Clinical Immunology, Division of Allergy, Immunology and Rheumatology, Department of Pediatrics, Faculty of Medicine Chulalongkorn University Bangkok Thailand; ^7^ Department of Internal Medicine, Division of Endocrinology Erasmus MC University Medical Center Rotterdam the Netherlands

**Keywords:** body mass index, IgG N‐glycosylation, immune aging, sex differences, smoking

## Abstract

**Background:**

*N*‐glycosylation drives immunoglobulin G (IgG) stability and function, directly affecting immune response. However, the effect of demographic and modifiable lifestyle factors on IgG *N*‐glycosylation in the general population remains poorly characterized.

**Methods:**

This study investigated the effects of age, sex, smoking, alcohol, body mass index (BMI), and time on IgG Fc *N*‐glycosylation patterns in the population‐based Rotterdam Study. IgG1–4 compositions were quantified by nanoflow liquid chromatography–electrospray ionization–mass spectrometry (nano–LC–ESI–MS) and expressed as percentages of the total signal. Cross‐sectional associations were assessed using the confounder‐adjusted multiple linear regression, while longitudinal changes were evaluated using the mixed effects models.

**Results:**

This study included 8242 participants (mean age = 64.2 years) with repeated measurements for 448 participants (mean follow‐up = 5.6 years). Age and BMI were associated with increased bisection and fucosylation (β = 0.007–0.030 SD/unit increase) and decreased galactosylation and sialylation (β = −0.007 to −0.057 SD). Women demonstrated higher bisection (β = 0.102–0.329 SD) across all subclasses, lower galactosylation, and sialylation in IgG_1_‐_3_ (β = −0.042 to −0.138 SD), but higher levels in IgG_4_ (β = 0.062–0.070 SD), and pronounced changes between 45 to 55 years. Smoking (β = 0.621 to 0.825 SD) and alcohol were associated with increased bisection, with a gradient effect in smoking status. With time, galactosylation and sialylation declined (β = −0.055 to −0.087 SD/year), while bisection increased (β = 0.030 to 0.050 SD/year).

**Conclusion:**

Aging, higher BMI, female sex, smoking, and heavy alcohol consumption are key determinants of IgG *N*‐glycome patterns, which are indicative of inflammation. Over time, glycan compositions demonstrate a gradual change toward a pro‐inflammatory phenotype in a sex‐related manner.

The effect of demographic and lifestyle factors on IgG *N*‐glycosylation, which determines IgG's inflammatory response, remains poorly characterized. Aging, higher BMI, female sex, smoking, and heavy alcohol consumption are determinants of IgG *N*‐glycome patterns, which are indicative of inflammation. Women exhibit a distinct change in IgG *N*‐glycosylation between age 45 and 55, a change that is absent in men. Repeated measurements confirm that *N*‐glycan compositions gradually evolve toward a pro‐inflammatory direction over time. BMI, Body‐mass index; IgG, Immunoglobulin G; Nano‐LC‐ESI‐MS, Nanoflow liquid chromatography–electrospray ionization–mass spectrometry.

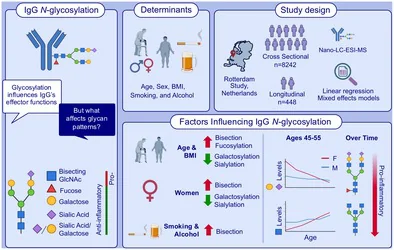

**Abbreviations**
ADCC: Antibody‐dependent cellular cytotoxicity
Bisecting GlcNAc: Bisecting *N*‐acetylglucosamine
Bisection: Bisecting *N*‐acetylglucosamine
BMI: Body Mass Index
CDC: Complement‐dependent cytotoxicity
DAGs: Directed acyclic graphs
Fc: fragment crystallizable
FDR: False discovery rate
ICC: Intraclass correlation coefficients
IgG: Immunoglobulin G
IgG_1‐4_
: Immunoglobulin G Subclasses
IVIg: Intravenous immunoglobulin
LC–MS: Liquid Chromatography–Mass Spectrometry
LMM: Linear mixed‐effects model
MICE: Multivariate Imputation by Chained Equations
RS: Rotterdam Study

## 1 Introduction

Immunoglobulin G (IgG), accounting for 75% of all antibodies in blood and extracellular fluid, is a vital component of the adaptive immune system. *N*‐glycosylation, an important post‐translational modification, drives IgG structure, stability, and function [1]. IgG *N*‐glycosylation involves the attachment of oligosaccharides (*N*‐glycans) to two predetermined sites on the fragment crystallizable (Fc) region of heavy chains of all four IgG subclasses (IgG_1–4_). Changes in *N*‐glycan composition affect how IgG interacts with cell surface receptors of innate immune cells, modulating pro‐inflammatory or anti‐inflammatory responses [2, 3], through altered Fc receptor engagement and complement activation [4, 5]. Studying how demographic and lifestyle factors regulate *N*‐glycosylation in the general population enhances our understanding of immune regulation and its impact on susceptibility to inflammation‐related disease.

Alterations of the IgG *N*‐glycome, the total set of *N*‐glycans, have been associated with premature aging conditions, [6, 7] as well as autoimmune disorders, cancer, and cardiovascular disease [1], conditions in which inflammatory processes are pivotal to disease etiology [8]. As such, variations in IgG *N*‐glycosylation are proposed not solely as an effect of disease states but rather as key contributors in the onset of disease. Indeed, a small number of studies have found that IgG *N*‐glycome changes precede rheumatoid arthritis [9, 10], cancer [11] and cardiovascular disease [12]. Subsequently, IgG *N*‐glycans have been identified as potential biomarkers and as features relevant to the development and optimization of glyco‐engineered therapeutics [5, 13, 14].

While limited studies have demonstrated associations between IgG glycans and determinants including age [15, 16, 17, 18, 19, 20, 21, 22, 23, 24], sex, [16, 17, 19, 20, 21, 22, 25] and lifestyle‐related factors [21, 23, 25, 26, 27, 28, 29, 30], interpretability for the general population is lacking. To date, associations have been observed in isolated populations [15, 17, 18, 26], disease‐specific cohorts, [16, 23, 24, 28] or hospital cohorts [20, 25, 27], or in studies with other limitations, including small sample size and the absence of formal regression analyses. Moreover, longitudinal research on the IgG *N*‐glycome is scarce [31].

The main objective of this study was to determine the effects of age, sex, smoking, alcohol consumption, and body mass index (BMI) on IgG *N*‐glycosylation patterns in an aging general population, focusing on glycan features associated with pro‐ and anti‐inflammatory immune states. In secondary analyses, we investigated changes in IgG *N*‐glycosylation patterns within the same individuals over time.

## 2 Methods

### 2.1 Study Design and Population

This study was part of the Rotterdam Study (RS), a large population‐based cohort study investigating the occurrence and etiology of age‐associated diseases in middle‐aged and older adults [32]. The RS has been previously described in detail [32]. Briefly, three independent cohorts (RSI, RSII, and RSIII) were included, comprising participants with available serum IgG data (*n* = 8411). RSI‐3 (1997–1999) and RSII (2000–2001) included participants aged ≥ 55 years, while RSIII (2006–2008) included participants aged ≥ 45 years. A random subset of 500 RSIII, 2 participants provided second IgG *N*‐glycosylation measurements 5–7 years later (2012–2014). Initial response rates in RSI, RSII, and RSIII were 78%, 67%, and 65%, respectively.

This study followed the Declaration of Helsinki and was approved by the Erasmus MC Medical Ethics Committee (registration number MEC 02.1015) and the Dutch Ministry of Health, Welfare and Sport (Population Screening Act WBO, license number 1071272–159521‐PG). All participants provided written informed consent.

### 2.2 IgG Fc *N*‐Glycosylation

IgG isolation and proteolytic generation of *N*‐glycopeptides have been performed as previously described [33], with minor modifications (see Supplementary Methods for the extended protocol). Briefly, IgG was captured from plasma using the protein G‐coated 96‐well plates, eluted with formic acid, and digested with trypsin overnight at 37°C. Peptides were analyzed using the liquid chromatography–mass spectrometry (LC–MS) [33]. Signal quality and quantity were evaluated based on predefined criteria [33] and the lowest quality fraction per IgG subclass was excluded. Due to the similar amino acid sequences in persons of European, Middle Eastern, or North African descent, IgG_2_ and IgG_3_ were collectively referred to as IgG_2/3_ [34]. Glycan compositions for IgG_1_, IgG_2/3_, and IgG_4_ were expressed as percentages of the total signal (Table S1). Five glycan structure groups, calculated by summing the relative areas of structurally similar glycans (Table S1), were derived for each subclass: core fucosylation, bisecting *N*‐acetylglucosamine (bisection), galactosylation, sialylation, and sialylation per galactose. Fucosylation in IgG_4_ could not be reliably measured due to the low abundance of IgG_4_ and interference from IgG_1_ glycoforms. Derived traits were classified as pro‐ or anti‐inflammatory based on recent literature (Supporting Information S1).

### 2.3 Potential Determinants

Potential determinants were selected based on biological plausibility, availability in the cohort, and a critical appraisal of current literature [15, 16, 17, 18, 19, 20, 21, 22, 23, 24, 25, 26, 27, 28, 29, 30], guided by directed acyclic graphs (DAGs) to minimize reverse causation bias (Supplementary Methods, Figure S1). Anthropometric measurements were collected during physical examination. BMI was computed (kg/m^2^), and waist circumference was measured in centimeters. Smoking was classified as current, former, or never, and alcohol consumption (g/day) as mild (0–10), moderate (10–20), or heavy (> 20) as per WHO guidelines [35]. Postmenopausal status was defined as the absence of menstruation for ≥ 12 consecutive months. Data on smoking, alcohol consumption, and menopause status were obtained through home interviews, except in RS II‐1, where alcohol consumption was assessed by questionnaire.

### 2.4 Statistical Analyses

Associations between the potential determinants and the Z‐standardized IgG *N*‐glycans were assessed cross‐sectionally using multiple linear regression models, with the potential determinant as exposure variable and each *N*‐glycan trait as outcome variable. Model 1 included age and sex; Model 2 additionally adjusted for modifiable lifestyle factors such as smoking and alcohol consumption; and the main Model 3 further included BMI. Non‐linear effects were assessed using the natural cubic splines. Additionally, analyses were stratified by sex, age groups (< 55 and ≥ 55 years), and menopausal status. Longitudinal changes in IgG *N*‐glycan traits were assessed using linear mixed‐effects models (LMMs) that included a random intercept adjusted according to Model 3, with time as the exposure variable and each *N*‐glycan trait as the outcome variable. Missing data were imputed and pooled according to the Rubin's rules. False discovery rate (FDR) correction for multiple testing (Benjamini–Hochberg, FDR < 0.05) was applied per IgG subclass in all individual compositions. Further model diagnostics and sensitivity analyses are described in Supporting Information S1. All statistical analyses were performed in R software version 4.4.1.

## 3 Results

We included 8242 participants with a mean age of 64.2 years, comprising 4711 women (57.2%) and 3531 men (42.8%), as shown in Table 1. Of the participants, 2757 were from RSI, 2245 from RSII, and 3240 from RSIII. The distribution of IgG *N*‐glycosylation traits varied widely (Figure S2), and correlations between traits were observed (Figure S4). Measurement precision was high, with technical variability being more than a magnitude lower than biological variation (Figure S3). After accounting for missing data, 8210 participants had IgG1 measurements, 8023 had IgG2/3, and 6130 had IgG4 measurements (Figure S5). In the subset with repeated measurements, 446 had repeated IgG1, 447 had IgG2/3, and 351 had IgG4. The subset baseline characteristics are shown in Table S2.

**TABLE 1:** Distribution of baseline potential determinants, stratified by sex.

	Men (*n* = 3531)	Women (*n* = 4711)	Overall (*n* = 8242)
Age (years)			
Mean (SD)	63.6 (9.27)	64.7 (10.2)	64.2 (9.83)
Median [Min, Max]	62.0 (45.6, 96.9)	62.4 (45.7, 106)	62.2 (45.6, 106)
Body mass index (kg/m^2^)			
Mean (SD)	27.2 (3.64)	27.5 (4.64)	27.3 (4.24)
Median [Min, Max]	26.8 (16.8, 46.7)	26.8 (12.6, 56.9)	26.8 (12.6, 56.9)
Smoking Status			
Never	729 (20.6%)	2020 (42.9%)	2749 (33.4%)
Former	2093 (59.3%)	1816 (38.5%)	3909 (47.4%)
Current	709 (20.1%)	875 (18.6%)	1584 (19.2%)
Alcohol Consumption			
Mild	1815 (51.4%)	3695 (78.4%)	5510 (66.9%)
Moderate	785 (22.2%)	664 (14.1%)	1449 (17.6%)
Heavy	931 (26.4%)	352 (7.5%)	1283 (15.6%)

Data quality assessment and model diagnostics for the linear regression models and LMM confirmed adherence to model assumptions. No batch or cohort effects were detected. The results were not confounded by ethnicity, and stratification under different ethnic backgrounds did not substantially change observations. An extensive description is found in Supporting Information S1.

The direction and magnitude of the effects in our primary model (Model 3) were consistent with those of Models 1 and 2. Table S3.1 summarizes the effects of the determinant factors on the four main IgG N‐glycan‐derived traits. Full results for all individual glycan compositions are presented in Table S3.2.

### 3.1 Determinants of the IgG *N*‐Glycome

#### 3.1.1 Age

Significant associations were found between age and glycan traits (Figure 1), with consistent effect directions across subclasses. Age was positively associated with bisect.ion (B = 0025 to 0.030, all *p* < 0.001) and fucosylation (B = 0.006 and 0.012, both *p* < 0.001) and negatively associated with galactosylation (B = −0.057 to −0.047, all *p* < 0.001) and sialylation (B = −0.050 to −0.040, all *p* < 0.001). All positively associated compositions, except H4N5F1 and H4N4F1S1, were agalactosylated. Mono‐ and digalactosylated compositions were negatively associated, with stronger effects for digalactosylated structures. The three most negatively (H5N4F1, H5N4F1S1, and H5N4F1S2) and positively (H3N4F1, H3N5F1, and H3N3F1) associated structures all contained fucose.

**FIGURE 1:**
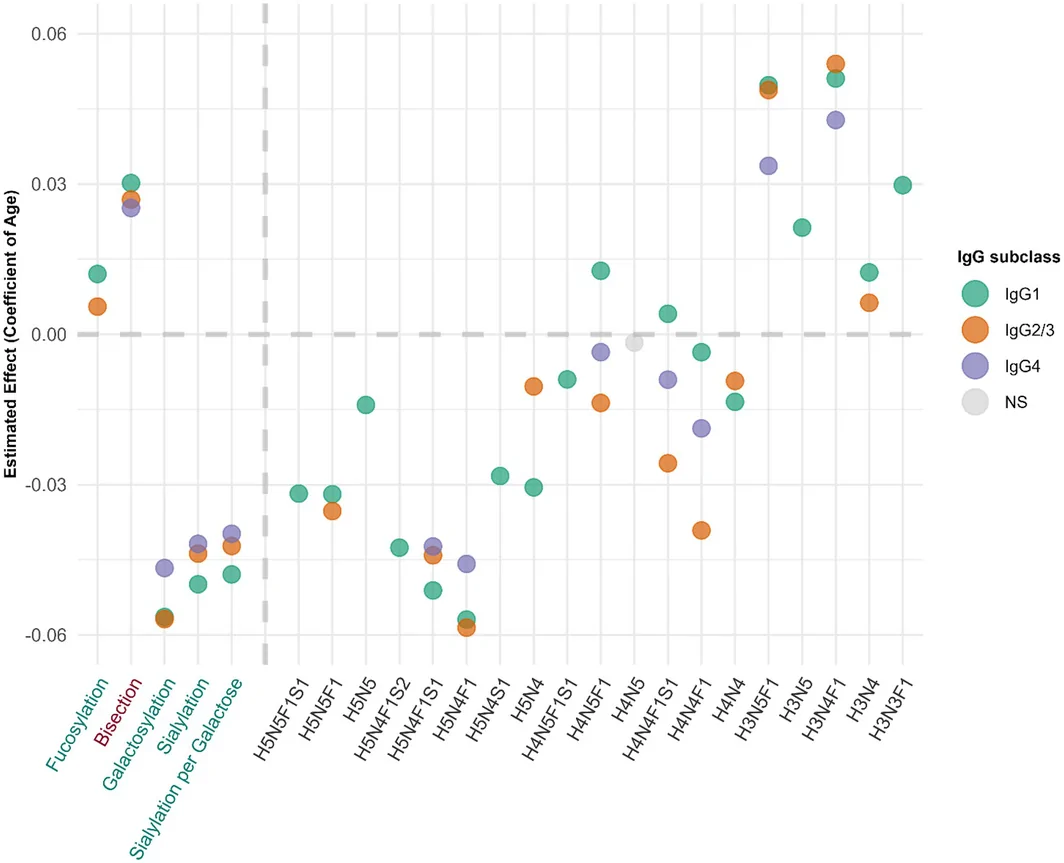
Effect of age on IgG *N*‐glycosylation traits and compositions. The figure displays estimated age effects (β estimates) on the y‐axis and individual IgG *N*‐glycosylation traits and compositions on the x‐axis. A vertical dashed line separates derived traits from individual glycan compositions. Data points are color‐coded by IgG subclass, with green representing IgG1, orange representing IgG2/3, and purple representing IgG4. Gray circles indicate traits that did not pass the significance threshold. Traits are additionally color‐coded by presumed inflammatory effect, with green indicating anti‐inflammatory traits and red indicating pro‐inflammatory traits. Glycan compositions are organized from most to least complex, with the most complex glycans generally associated with anti‐inflammatory responses and the least complex glycans linked to pro‐inflammatory outcomes. All estimates are adjusted for mediators: sex, smoking, alcohol consumption, and BMI.

#### 3.1.2 Sex

Significant sex‐specific differences were observed in glycan traits across IgG subclasses (Figure 2). Compared to men, women exhibited higher levels of bisection across all subclasses, with the largest effect in IgG_4_ (B = 0.329), followed by IgG_2/3_ (B = 0.212) and IgG_1_ (B = 0.102), with all *p* < 0.001. This pattern was further demonstrated in H4N4F1 (IgG_4_ B = −0.205, IgG_2/3_ B = −0.115, both *p* < 0.001) and H4N5F1 (IgG_1_ B = 0.138, IgG_2/3_ B = 0.120, IgG_4_ B = 0.282, all *p* < 0.001), which differ by bisection and showed strong opposing effects. Women demonstrated lower percentages of galactosylation, sialylation, and sialylation per galactose in IgG_1_ (B = −0.042 to −0.067) and IgG_2/3_ (B = −0.112 to −0.145), but higher percentages in IgG_4_ (B = 0.068 and 0.061, respectively), with all *p* < 0.05. This opposite effect direction in IgG_4_ was also reflected in specific glycan structures, as shown in Figure 2.

**FIGURE 2:**
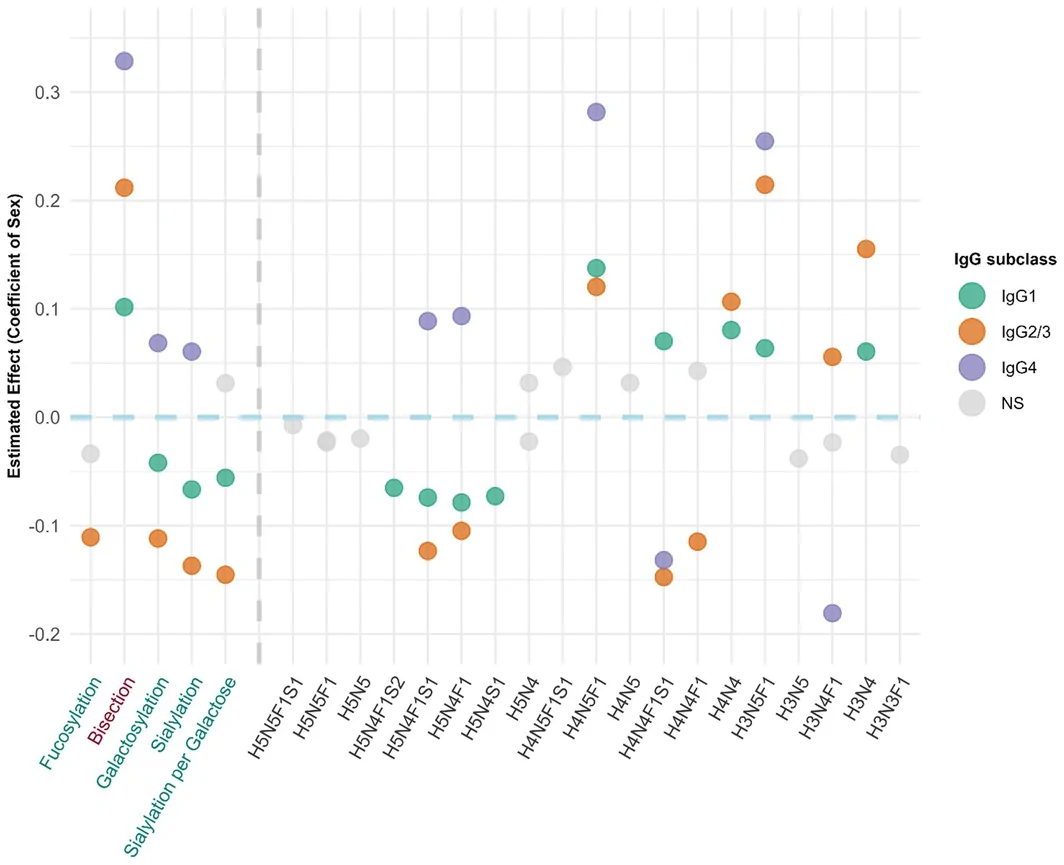
Effect of sex on IgG *N*‐glycosylation traits and compositions. The figure presents the estimated effects of female sex (compared to male) on IgG *N*‐glycosylation traits and compositions, with β estimates on the y‐axis and individual traits on the x‐axis. A vertical dashed line separates derived traits from individual glycan compositions. Data points are color‐coded by IgG subclass, with green representing IgG1, orange representing IgG2/3, and purple representing IgG4. Gray circles indicate traits that did not pass the significance threshold. Traits are additionally color‐coded by presumed inflammatory effect, with green indicating anti‐inflammatory traits and red indicating pro‐inflammatory traits. Glycan compositions are organized from most to least complex, with the most complex glycans generally associated with anti‐inflammatory responses and the least complex glycans linked to pro‐inflammatory outcomes. All estimates are adjusted for mediators like age, smoking, alcohol consumption, and BMI.

#### 3.1.3 Sex and Age Interaction

The interaction effect of sex and age on IgG glycans was assessed using the models with and without restricted cubic splines for age. The linear models generally showed no significant interaction, whereas the spline models revealed interaction effects between ages 45–55 (Figure S6). In this age group (740 women and 576 men), sex‐specific glycan trajectories diverged significantly, with women showing decreasing galactosylation and sialylation and increasing bisection, while men exhibited minimal changes (Figure S6). After age 55, interaction effects diminished. In addition, the associations of all determinants were consistent in peri‐ and post‐menopausal women (see Figure S7).

#### 3.1.4 BMI

Higher BMI was associated with lower galactosylation (B = −0.034, *p* < 0.001), sialylation (B = −0.025, *p* < 0.001), and sialylation per galactose (B = −0.022, *p* < 0.001) in IgG_1_ (Figure 3). BMI was positively associated with bisection in IgG_1_ (B = 0.019, *p* < 0.001) and IgG_4_ (B = 0.016, *p* < 0.001) and with fucosylation across subclasses (IgG_1_ B = 0.015 and IgG_2/3_ B = 0.007, *p* < 0.001 and *p* = 0.01). Results were consistent when replacing BMI with waist circumference. Overall, BMI and age showed similar effect directions, while there was no considerable correlation between BMI and age, and findings for age did not substantially change when BMI was added to the model (Supporting Information S1).

**FIGURE 3:**
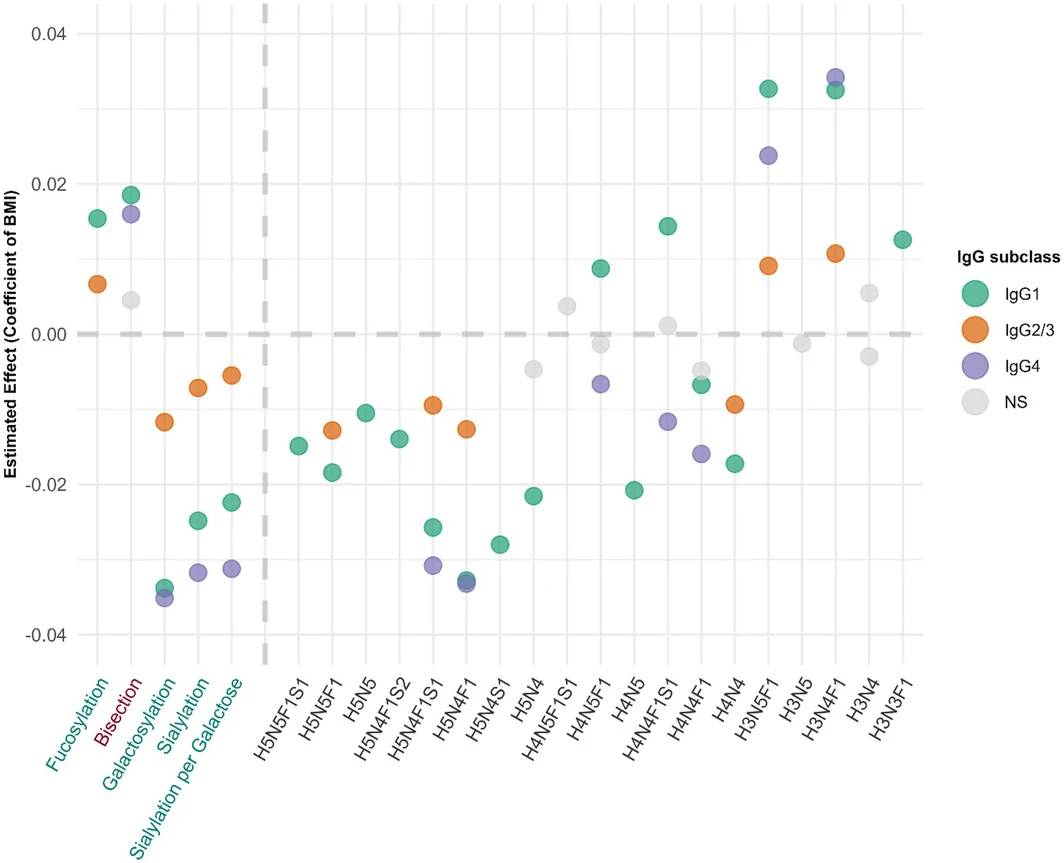
Effect of BMI on IgG *N*‐glycosylation traits and compositions. The figure presents the estimated effects of higher BMI on IgG *N*‐glycosylation traits and compositions, with β estimates on the y‐axis and individual traits on the x‐axis. A vertical dashed line separates derived traits from individual glycan compositions. Data points are color‐coded by IgG subclass, with green representing IgG1, orange representing IgG2/3, and purple representing IgG4. Gray circles indicate traits that did not pass the significance threshold. Traits are additionally color‐coded by presumed inflammatory effect, with green indicating anti‐inflammatory traits and red indicating pro‐inflammatory traits. Glycan compositions are organized from most to least complex, with the most complex glycans generally associated with anti‐inflammatory responses and the least complex glycans linked to pro‐inflammatory outcomes. All estimates are adjusted for confounders age, sex, smoking, and alcohol consumption.

#### 3.1.5 Smoking

Compared to non‐smokers, current and former smokers exhibited similar effect directions, with stronger effects in current smokers (Figure 4). Current (B = 0.619 to 0.822, all *p* < 0.001) and former smoking (B = 0.146 to 0.180, all *p* < 0.001) were associated with higher bisection across all subclasses. Bisected glycans H3N5F1 and H4N5F1 showed the strongest positive associations, while non‐bisected counterparts (H3N4F1, H4N4F1) were most negative. Fucosylation was lower in current (IgG1 B = −0.182, IgG2/3 B = −0.315, *p* < 0.001) and former smokers (IgG1 B = −0.070, IgG2/3 B = −0.058, *p* = 0.006 and *p* = 0.026). Galactosylation, sialylation, and sialylation per galactose exhibited subclass‐specific effects: higher in IgG2/3 (B = 0.055 to 0.083, all *p* < 0.05), with the exception of sialylation per galactose in current smokers, but lower in IgG1 (B = −0.086 to −0.078, all *p* < 0.01) and IgG4 (B = −0.068 to −0.116, all *p* < 0.05).

**FIGURE 4:**
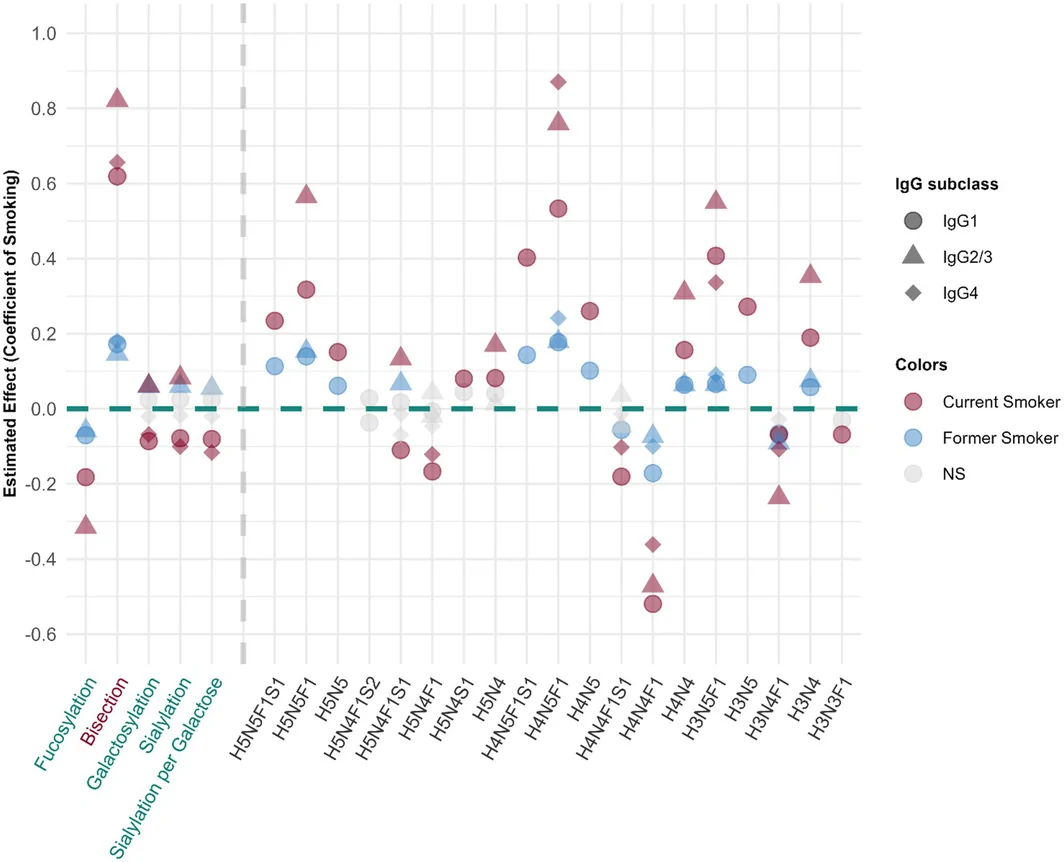
Effect of smoking on IgG *N*‐glycosylation traits and compositions. The figure presents the estimated effects of current and former smoking (compared to never smoking) on IgG *N*‐glycosylation traits and compositions, with β estimates on the y‐axis and individual traits on the x‐axis. A vertical dashed line separates derived traits from individual glycan compositions. Data points are distinguished by shape, with circles representing IgG1, triangles representing IgG2/3, and diamonds representing IgG4. Points are further color‐coded by smoking status, with red indicating current smokers, blue indicating former smokers, and gray indicating traits that did not pass the significance threshold. Glycan compositions are organized from most to least complex, with the most complex glycans generally associated with anti‐inflammatory responses and the least complex glycans linked to pro‐inflammatory outcomes. All estimates are adjusted for confounders age, sex, alcohol consumption, and mediator BMI.

#### 3.1.6 Alcohol

Heavy alcohol consumption was associated with increased bisection in all subclasses (B = 0.099 to 0.110, all *p* < 0.01), as well as higher sialylation (B = 0.111, *p* = 0.002) and sialylation per galactose (B = 0.117, *p* = 0.001) in IgG_2/3_ (Figure 5). Moderate and heavy alcohol consumption were also associated with lower levels of fucosylation in IgG_2/3_ (B = −0.068, *p* = 0.025 and B = −0.125, *p* = 0.001, respectively).

**FIGURE 5:**
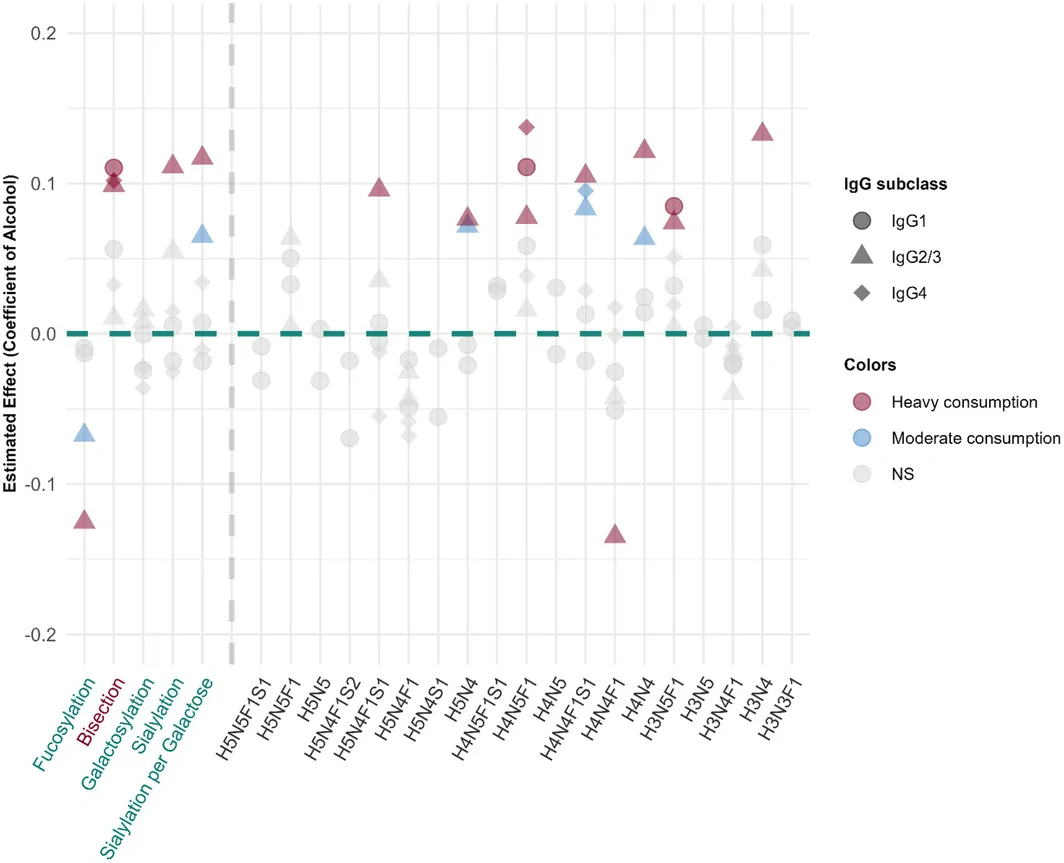
Effect of alcohol consumption on IgG *N*‐glycosylation traits and compositions. The figure presents the estimated effects of heavy and moderate alcohol consumption (compared to mild consumption) on IgG N‐glycosylation traits and compositions, with β estimates on the y‐axis and individual traits on the x‐axis. A vertical dashed line separates derived traits from individual glycan compositions. Data points are distinguished by shape, with circles representing IgG1, triangles representing IgG2/3, and diamonds representing IgG4. Points are further color‐coded by consumption level, with red indicating heavy consumption, blue indicating moderate consumption, and gray indicating traits that did not pass the significance threshold. Glycan compositions are organized from most to least complex, with the most complex glycans generally associated with anti‐inflammatory responses and the least complex glycans linked to pro‐inflammatory outcomes. All estimates are adjusted for confounders age, sex, smoking, and mediator BMI.

#### 3.1.7 Longitudinal Age‐Related Changes in IgG *N*‐Glycosylation

Longitudinal trajectories of IgG *N*‐glycan traits were assessed (Figure S8, Table S4 and Table S5). Galactosylation and sialylation declined across all IgG subclasses. IgG_4_ showed the steepest galactosylation decline (B = −0.070 SD/year, *p* < 0.001). Sialylation, including sialylation per galactose, also decreased, with IgG_1_ and IgG4 showing a notable reduction (B = −0.056 SD/year and – 0.087 SD/year, *p* < 0.001). Conversely, bisection increased across all subclasses, with the steepest rise in IgG_1_ (B = 0.050 SD/year, *p* < 0.001). Fucosylation varied by subclass: fucosylation remained stable in IgG_1_ (*p* > 0.05), whereas IgG_2/3_ fucosylation displayed a decline in time (B = −0.081 SD/year, *p* < 0.001). Despite these average time effects, the ICCs ranged from 0.81 to 0.93 (Table S6), indicating that traits exhibit strong stability within individuals.

## 4 Discussion

To date, this is the largest study on determinants of IgG *N*‐glycosylation in an aging general population, uniquely incorporating repeated measures to assess longitudinal changes. We identified aging, higher BMI, female sex, smoking, and heavy alcohol consumption as key determinants of the IgG *N*‐glycome, leading to alterations reflecting a more pro‐inflammatory state of IgG. Aging and higher BMI showed a similar IgG glycome signature, with subtle variations across subclasses. Sex‐specific differences were observed, with glycan trajectories divergent in midlife but more closely aligned at older ages. Despite the stability of the IgG *N*‐glycome, we observed gradual glycome changes over time, with cross‐sectional aging effects similar to longitudinal intra‐individual alterations.

Glycosylation drives IgG function by altering receptor interactions, modulating pro‐inflammatory or anti‐inflammatory responses [1]. Although glycosylation affects multiple pathways, evidence from in vitro and in vivo studies suggests that afucosylation and bisection promote pro‐inflammatory responses, whereas galactosylation and sialylation are associated with anti‐inflammatory effects [1, 36, 37]. A more detailed rationale for these functional classifications is provided in Supplementary Methods.

Our study confirms that aging is associated with decreased galactosylation and sialylation, alongside increased bisection across all IgG subclasses. Cross‐sectional and longitudinal observations are closely aligned, reinforcing the robustness of observed patterns, with fucosylation being the only trait showing a discrepancy. As longitudinal analyses minimize confounding due to stable individual characteristics, we consider the longitudinal effects more valid, suggesting fucosylation likely decreases with age in IgG_2/3_, but remains stable in IgG_1_. Our findings are consistent with previous subclass‐specific and total IgG studies in diverse populations [15, 17, 18, 20, 22]. The observed glycome changes may reflect immunosenescence, the age‐related decline in immune function that leads to increased infection susceptibility and age‐related diseases, and is associated with inflammaging [38]. In accordance, our findings suggest a gradual shift toward a more pro‐inflammatory profile of IgG. These *N*‐glycan changes could contribute to a self‐perpetuating loop where inflammation both drives and results from immune decline [1, 38]. In addition to immunosenescence, age‐related changes in IgG *N*‐glycome reflect the accumulation of exposure to environmental factors and disease states during the passing of time [39], various of which have been associated with IgG glycosylation alterations [1].

Our study is the first to confirm sex‐specific IgG glycan traits. Between ages 45 and 55, women demonstrated a faster decline in galactosylation and sialylation, combined with a sharper rise in bisection compared to men, likely driven by hormonal changes accompanying menopause. Estrogen directly influences glycosylation enzymes [40, 41, 42, 43], and estrogen loss reduces galactose and increases fucose, as seen in leuprolide‐induced menopause, with effects reversible by estrogen supplementation [44]. Similarly, Deriš et al. [45]. observed an accelerated decline in galactosylation and sialylation during menopause in total IgG. Although age trajectories converged after age 55, distinct sex differences remained evident in our predominantly post‐menopausal population, consistent with findings by Chen et al. [16] in the elderly. Higher testosterone and estradiol levels in men compared to postmenopausal women likely contribute to these persistent differences [44]. Interestingly, IgG_4_ glycosylation patterns were generally opposite to those in IgG_1‐3_, though the functional significance remains unclear to date [46]. Our observations suggest a more pro‐inflammatory IgG profile in peri‐ and post‐menopausal women (Figure S7), potentially contributing to increased susceptibility of inflammation‐mediated conditions, including cardiovascular disease [47], type II diabetes, [12] and autoimmune diseases [10]. These observations underscore the need for further research to clarify the role of sex‐specific immune function in the pathogenesis of these diseases.

Higher BMI was negatively associated with galactosylation and sialic acid and positively associated with fucosylation and bisection, consistent with earlier studies regarding BMI and body fat distribution [26, 48]. Our findings suggest that higher BMI contributes to a more pro‐inflammatory IgG response. This is consistent with the chronic low‐grade inflammation observed in obesity, potentially due to the secretion of inflammatory adipokines by adipose tissue [49], which is mechanistically linked to insulin resistance, type II diabetes, metabolic syndrome, and cardiovascular disease [8, 50, 51]. Similar associations observed for BMI and waist circumference indicate that fat tissue, rather than weight alone, seems to be a driving factor in this association. Strikingly, BMI and age showed comparable glycome signatures, with all significant associations sharing similar effect directions. Our findings suggest that age and BMI affect the humoral immune system in similar ways, independent of each other, and that their effect on immune dysfunction and, consequently, disease susceptibility may be comparable.

Our findings reveal a smoking‐related IgG glycan shift. A strong gradient of increased bisection and decreased fucosylation was observed, with the strongest effects in current smokers and weaker but persistent effects in former smokers compared to never smokers. Cigarette smoke generates oxidative stress, which could disrupt glycosylation [52, 53] and is thought to upregulate MGAT3, an enzyme that catalyzes bisection, by hypomethylation [54, 55, 56]. Consequently, active smoking might amplify inflammatory potential, while residual effects diminish after cessation, aligning with previous research [28, 57]. Our observations suggest that the smoking‐induced immunomodulatory effects may persist over time, but are, at least partly, reversible. The observed effects of alcohol were relatively small compared to other determinants, and the effect of alcohol consumption on IgG function therefore seems more limited.

This study's strengths include its large, population‐based cohort, enhancing generalizability, and longitudinal measurements, enabling assessment of temporal dynamics in IgG *N*‐glycosylation. Careful assessment of determinants, including non‐linearity, stratification, and sensitivity analyses, supported the robustness of findings. Mass spectrometry provided high‐resolution data and enabled subclass‐specific evaluation of glycosylation patterns. Limitations include the predominantly European population. Nonetheless, sensitivity analyses showed no major differences among different ethnicities (Supporting Information S1). The low abundance of serum IgG_4_ increased variability, resulting in a relatively high percentage of missing values (25.6%), potentially introducing selection bias. Yet, this is the first study to report IgG4 glycosylation patterns in a general population.

Further research should aim to clarify the functional significance of subclass‐specific glycosylation changes and their role in immune regulation. Moreover, identifying these early changes in population‐based cohorts is essential for understanding their role in disease development.

## 5 Conclusions

To conclude, we identified aging, higher BMI, female sex, smoking, and heavy alcohol consumption as key determinants of the IgG Fc *N*‐glycome, leading to alterations indicative of increased inflammation. Over time, the *N*‐glycome exhibits a gradual, sex‐related shift toward a more pro‐inflammatory IgG profile. Our work offers valuable insights into potential confounding factors for future glycosylation research, underscores the potentially reversible effects of modifiable determinants, and suggests factors that may contribute to susceptibility to inflammation‐related diseases.

## Author Contributions

Samantha Leonard and Ralph Heijmans contributed equally and share first authorship; they performed all statistical analyses and drafted the manuscript. Samantha Leonard, Ralph Heijmans, Layal Chaker, Virgil Dalm, and Manfred Wuhrer contributed to study design and data interpretation. Manfred Wuhrer, Jan Nouta, Steinar Gijze, and David Falck conducted IgG *N*‐glycan measurements and preprocessing. All authors, including P. Martin van Hagen, Robin Peeters, and Silvan Licher, critically revised the manuscript. Layal Chaker and Virgil Dalm supervised the study; Layal Chaker and Silvan Licher acquired funding. To ensure data integrity, Samantha Leonard, Ralph Heijmans, Manfred Wuhrer, Jan Nouta, Steinar Gijze, and David Falck directly accessed and verified the underlying data reported in the manuscript. All authors approved the final manuscript.

## Funding

Layal Chaker, Samantha Leonard, and Silvan Licher were supported by ZonMW grant [10430362220003]. The Rotterdam Study is funded by Erasmus Medical Center and Erasmus University, Rotterdam, Netherlands Organization for the Health Research and Development (ZonMw), the Research Institute for Diseases in the Elderly (RIDE), the Ministry of Education, Culture and Science, the Ministry for Health, Welfare and Sports, the European Commission (DG XII), and the Municipality of Rotterdam. The funding sponsors and organizations did not influence study design, collection, analysis, and interpretation of data; nor the decision to submit the article for publication.

## Conflicts of Interest

Layal Chaker, Samantha Leonard, and Silvan Licher were supported by ZonMW grant [10430362220003]. The Rotterdam Study is funded by Erasmus Medical Center and Erasmus University, Rotterdam, Netherlands Organization for the Health Research and Development (ZonMw), the Research Institute for Diseases in the Elderly (RIDE), the Ministry of Education, Culture and Science, the Ministry for Health, Welfare and Sports, the European Commission (DG XII), and the Municipality of Rotterdam.

## Supporting information


**Table S1:** Glycan compositions of the IgG *N*‐glycome.
**Table S2:** Repeated Measures Baseline Characteristics.
**Table S3:** 1. Summary of Model 3 Results.
**Table S4:** Effect of binary time on IgG glycosylation.
**Table S5:** Effect of continuous time (years since baseline) on IgG glycosylation.
**Table S6:** Intraclass Correlation Coefficients.
**Figure S1:** Directed Acyclic Graph.
**Figure S2:** Glycan Trait Distribution.
**Figure S3:** Assay Performance.
**Figure S4:** Correlation matrix.
**Figure S5:** Inclusion and Exclusion of Participants.
**Figure S6:** Age‐Sex Interaction Effects on IgG Glycans.
**Figure S7:** Analyses Stratified by Menopause Status.
**Figure S8:** Longitudinal Changes in IgG Glycans.
Supplementary Methods.
Supplementary Results.
Supplementary Tables.
Supplementary References.

Table S3:2: Summary of Model 1, Model 2, and Model 3 Results.

## Data Availability

Data can be obtained upon request. Requests should be directed towards the management team of the Rotterdam Study (secretariat.epi@erasmusmc.nl) and the corresponding author. Due to restrictions based on privacy regulations and informed consent of the participants, data cannot be made freely available in a public repository.
