# Supplementary material for: Determinants of Immunoglobulin‐G N‐Glycan Patterns in the Aging General Population
Source: Allergy. 2026 Apr 30;81(11):4065–74. doi: 10.1111/all.70361 (PMC13753674; doi:10.1111/all.70361)
Supplement: Supplementary file 1 [file ALL-81-4065-s001.docx]

**Determinants of Immunoglobulin G *N-*Glycan Patterns in the Aging General Population**

Samantha Leonard, MSc ^a,b,1^, Ralph Heijmans, MD ^a,b,1^, Virgil A.S.H. Dalm, MD, PhD ^a,c^, Silvan Licher, MD, PhD ^b,d^, Jan Nouta, BSc ^e^, Steinar Gijze, BSc ^e^, David Falck, PhD ^e^, P. Martin van Hagen, MD, PhD ^a,c,f^, Robin Peeters, MD, PhD, ^g^, Manfred Wuhrer, PhD ^e^, Layal Chaker, MD, PhD ^b,g^

^a^ Department of Internal Medicine, Division of Allergy & Clinical Immunology, Erasmus MC University Medical Center, Rotterdam, the Netherlands

^b^ Department of Epidemiology, Erasmus MC University Medical Center, Rotterdam, the Netherlands

^c^ Department of Immunology, Erasmus MC University Medical Center, Rotterdam, the Netherlands

^d^ Department of Primary Care, Erasmus MC University Medical Center, Rotterdam, the Netherlands

^e^ Center for Proteomics and Metabolomics, Leiden University Medical Center, Leiden, The Netherlands

^f^ Center of Excellence for Allergy and Clinical Immunology, Division of Allergy, Immunology and Rheumatology, Department of Pediatrics, Faculty of Medicine, Chulalongkorn University, Bangkok, Thailand

^g^ Department of Internal Medicine, Division of Endocrinology, Erasmus MC University Medical Center, Rotterdam, the Netherlands

^1^ S.L. and R.H. contributed equally to this work

**Corresponding author:**

Layal Chaker

[l.chaker@erasmusmc.nl](mailto:l.chaker@erasmusmc.nl)

Department of Internal Medicine, Erasmus MC – University Medical Center Rotterdam, Doctor Molewaterplein 40, 3015 GD, Rotterdam, The Netherlands

**Supplementary Material – Table of Contents**

1. **Supplementary Methods**
2. **Supplementary Results**
3. **Supplementary Tables**
   a. Table S1: Glycan compositions of the IgG *N*-glycome
   b. Table S2: Repeated Measures Baseline Characteristics
   c. Table S3.1: Summary of Model 3 Results
   d. Table S4: Effect of binary time on IgG glycosylation
   e. Table S5: Effect of continuous time (years since baseline) on IgG glycosylation
   f. Table S6: Intraclass Correlation Coefficients
4. **Supplementary Figures**
   a. Figure S1: Directed Acyclic Graph
   b. Figure S2: Glycan Trait Distribution
   c. Figure S3: Assay Performance
   d. Figure S4: Correlation matrix
   e. Figure S5: Inclusion and Exclusion of Participants
   f. Figure S6: Age-Sex Interaction Effects on IgG Glycans
   g. Figures S7: Analyses Stratified by Menopause Status
   h. Figures S8: Longitudinal Changes in IgG Glycans
5. **Supplementary References**

# **1. Supplementary Methods**

**Detailed IgG *N*-glycan Extraction**

IgG isolation and proteolytic generation of *N*-glycopeptides have been performed as described before [1], with minor modifications. In short, 96-well Maxisorp plates were coated with Protein G (5 µg/ml in PBS; Thermo #101201) for 1h at 37°C. Plates were washed three times with PBS-T before adding 2 µl of plasma diluted in 50 µl of PBS-T followed by a 1 h incubation at room temperature for IgG capturing. After washes, IgG was eluted with 100 mM formic acid, transferred to V-bottom plates and dried. For proteolytic cleavage, dried IgG samples were reconstituted in 50mM ammonium bicarbonate (ABC), and trypsin cleavage mix was added. After overnight incubation at 37 °C, samples were stored at -20 °C until liquid chromatography–mass spectrometry (LC–MS) readout [1]. Signal quality and quantity were evaluated based on predefined criteria [1] and the lowest quality fraction per IgG subclass was excluded to ensure data quality. Due to the similar amino acid sequences in persons of European, Middle Eastern, or North African descent, IgG_2_ and IgG_3_ could not be distinguished and were collectively referred to as IgG_2/3_ [2]. Glycan compositions for IgG_1_, IgG_2/3_, and IgG_4_ were expressed as percentages of the total signal (Supplemental Table S1). Five glycan structure groups, calculated by summing the relative areas of structurally similar glycans (Supplemental Table S1), were derived for each subclass: core fucosylation, bisecting *N*-acetylglucosamine (bisection), galactosylation, sialylation and sialylation per galactose. Fucosylation in IgG_4_ could not be reliably measured due to the relatively low abundance of IgG_4_ and the interference of IgG_1_ fucosylated glycoforms with IgG_4_ afucosylated glycoforms.

**Presumed Function of IgG *N*-glycans**

Based on recent literature reviews of in vitro cell receptor studies and in vivo rodent studies on IgG N-glycosylation [3-6], the presumed (simplified) function of each measured IgG N-glycan derived trait was determined and shown in Figure 1-5. Here, further considerations are addressed.

Glycosylation drives IgG function by changing cell surface receptor interactions, modulating pro-inflammatory or anti-inflammatory responses depending on glycan composition [4]. However, the four main glycan features—core fucose, bisecting GlcNAc (bisection), galactose and sialic acid—cannot be definitively classified as entirely pro- or anti-inflammatory, as IgG *N*-glycosylation, affects various inflammatory and regulatory pathways simultaneously, not rarely in a conflicting manner [4, 6]. Nevertheless, in vitro cell receptor and in vivo rodent studies have given insight into how these glycan features influence overall inflammatory status.

For instance, the lack of fucose is associated with a pro-inflammatory effect due to improved FcγRIII binding followed by increased antibody-dependent cellular cytotoxicity (ADCC) in all IgG subclasses [3, 4, 7], a mechanism leveraged in therapeutic monoclonal antibodies [3, 8]. Similarly, the addition of bisecting N-acetylglucosamine (bisection) has been found to be associated with a pro-inflammatory effect in both fucosylated and afucosylated structures due to improved FcγRIII binding, although evidence is conflicting [3, 6, 7]. Galactosylation and sialylation are associated with anti-inflammatory effects [3, 4]. In rodent models, both modifications enhance the anti-inflammatory action of intravenous immunoglobulin (IVIg) therapy [3, 9, 10]. In arthritic mice, IgG autoantibodies lacking galactose show increased disease-transferring ability [11], while those containing sialic acid suppressed arthritis development [12]. However, in vitro studies also link galactose and sialic acid to increased ADCC and complement-dependent cytotoxicity (CDC) [4, 6, 7], suggesting that exact mechanisms underlying their anti-inflammatory effects remain unclear.

Considering this information, the addition of fucose was considered anti-inflammatory, while afucosylation was considered pro-inflammatory. Presence of bisected *N*-acetylglucosamine was considered pro-inflammatory. Both the addition of galactose and sialic acid were considered anti-inflammatory. Subsequently, sialic acid per galactose (S/G) was also considered anti-inflammatory.

## **Potential determinants**

A comprehensive literature search was conducted through a critical appraisal of literature[13] to identify potential determinants. Relevant studies were identified using PubMed using the following search term:
("Immunoglobulin G"[Mesh]OR IgG [tiab]) AND ("Glycosylation"[Mesh] OR Glycan* [tiab]) AND (Epidemiologic Studies [Mesh] OR epidemiologic* [tiab] OR cohort* [tiab] OR cross-section* [tiab] OR case-control*[tiab] OR population[tiab])

Articles were screened based on relevance to IgG *N*-glycosylation and its determining factors, focusing on observational evidence from human studies, published in English until July 5 2024. Directed acyclic graphs (DAGs) were created to display potential causal relationships and to help identify mediators and confounders. To limit reverse causation bias, only covariates with a clear direction of cause-and-effect relationship were selected in this study.

**Detailed Statistical Analysis**

The associations between potential determinants and IgG *N*-glycan compositions and glycan structure groups were assessed by multiple linear regression models. Glycan compositions and structure groups were Z-standardized for comparison. Model 1 included non-modifiable factors age and sex; Model 2 included additional adjustments for modifiable lifestyle factors smoking and alcohol consumption; and our main model Model 3 additionally accounted for BMI. BMI was separately included into a model as 1) it was regarded as a mediator rather than a confounder alone and 2) the possibility of reverse causation for BMI could not be excluded. Non-linear effects were assessed by natural cubic splines with three degrees of freedom. Additionally, analyses were stratified by sex and age groups (<55 years and ≥55 years). A number of sensitivity analyses were conducted, exploring differences in batch, cohort, ethnicity, and BMI/waist circumference, detailed in Supplementary Material 1. To assess the crude relationships between glycosylation traits across IgG subclasses 1, 2/3, and 4, correlations were estimated and summarized in a correlation matrix (Supplementary Fig. S4). Longitudinal changes in IgG *N*-glycan traits were assessed using a linear mixed-effects model (LMM) to account for repeated measurements within individuals and was adjusted according to Model 3. Intraclass correlation coefficients (ICCs) were calculated from LMM models to assess within-individual stability. Missing data was imputed using Multivariate Imputation by Chained Equations (MICE, 10 imputations, 50 iterations) and pooled according to Rubin's rules. False discovery rate (FDR) correction for multiple testing was applied per IgG subclass in all individual compositions.

**Sensitivity Analysis of Batch, Cohort, BMI, Ethnicity and Menopause Effects**

Linear mixed models (LMMs) were employed to assess batch and cohort effects in IgG *N*-glycan traits, adjusting for age, sex, smoking, alcohol consumption, and BMI. Batch effects were modeled with a random intercept, with model fit evaluated using Bayesian Information Criterion (BIC) and Akaike Information Criterion (AIC), and likelihood ratio tests (LRT). Intraclass correlation coefficients (ICC) were calculated to compare models and quantify the variance attributable to batch effects. Cohort effects were assessed similarly, using a random intercept for cohort (Studie) to capture baseline differences across groups, with model fit assessed using BIC, AIC, LRT, and ICC.

The effect of BMI on regression models for IgG glycan traits was assessed by comparing two models: Model 2 (excluding BMI) and Model 3 (including BMI). ANOVA was used to evaluate differences in model fit between the two models. Additionally, changes in age-related coefficients were analyzed to determine the impact of including BMI on glycan trait associations. To further assess the effect of BMI, a secondary analysis of model 3 was examined, in which BMI was substituted by waist circumference.

Ethnicity was classified using the ASCCEG system, with participants grouped as either 'European, Middle Eastern, or North African' or 'other' based on IgG subclass sequence differences. In persons with a European, Middle Eastern, or North African ancestry (‘Caucasians’), IgG2 and IgG3 share similar amino acid sequences and can therefore not be distinguished in mass spectrometric analysis. However, in persons from Asian descent, IgG3 shares a common sequence with IgG4.[2] To account for this potential measurement error and to assess generalizability and confounding by ethnicity, stratified analyses and models including these classifications were conducted.

To assess the potential influence of menopausal status on our main cross-sectional analyses, stratified multiple linear regression analyses were performed for determinants age, BMI, smoking and alcohol consumption. The analyses were restricted to women from the RSIII cohort, which includes participants aged ≥45 years and therefore comprises both premenopausal and postmenopausal women.

**Diagnostics for Cross-Sectional and Longitudinal Models**

Assumptions for linear regression were evaluated through residual and QQ plots to examine heteroscedasticity and normality. Collinearity of covariates was checked by variance inflation factors (VIFs). Non-linearity for age and BMI was assessed using restricted cubic splines with 3 degrees of freedom. For the longitudinal analysis, residual diagnostics, including Q-Q plots, were used to validate model assumptions. Model selection criteria included AIC, BIC and LRT to confirm the necessity of random effects.

**Stability assessment**

Besides identification of batch and cohort effects, Intraclass Correlation Coefficients (ICCs) were also calculated to assess intra-individual stability of the *N*-glycan derived traits and compositions over time. The unadjusted ICCs were calculated based on a random-intercept-only LMM model, which reflects the proportion of total variance attributable to between-individual differences. The adjusted ICC accounts for the observed average change over time by including time as a fixed effect in LMM models.

# **2. Supplementary Results**

**Diagnostics for Cross-Sectional and Longitudinal Models**

For the cross sectional analysis, model 3 generally showed no major deviations from model assumptions. Residual plots demonstrated homoscedasticity, with no discernible patterns in residuals versus fitted values, except for clustering of IgG_1_ and IgG_2/3_ H4N4F1 and IgG_4_ H4N5F1. Adding an age-sex interaction resolved clustering for IgG_1_ H4N4F1. For IgG_2/3_ and IgG_4_ H4N5F1, clustering appeared linked to smoking-sex subgroups; removing smoking resolved this issue without notable changes in other effect sizes. Q-Q plots confirmed approximately normal distributions, with minor tail deviations for some glycan traits, supporting overall model validity.

Diagnostic analyses supported the robustness of the LMM models. Random intercepts captured significant inter-individual variability in baseline glycan levels, with variance estimates ranging from 0·47 and 0·90. Observed versus fitted values showed strong alignment, with minor deviations for specific traits like IgG_1_ H3N3F1 due to high leverage outliers. Residual diagnostics confirmed approximate normality, and model performance improved significantly with random intercepts compared to a fixed-effects model (ΔBIC -57·37 to -882·24), with all LRT’s further indicating strong random effects (all P < 0·0001).

**Sensitivity analyses**

The correlation matrix shown in Supplementary Fig. S4 reveals several key relationships of glycosylation among the IgG subclasses. Overall, several glycosylation traits show consistent cross-subclass correlations. For example, IgG1 bisection is positively correlated with IgG2/3 bisection and IgG4 bisection, and IgG1 galactosylation is positively associated with IgG2/3 and IgG4 galactosylation as well as sialylation traits across subclasses. In contrast, bisection shows negative correlations with galactosylation and sialylation, supporting the opposing functional directionality of these glycan features. Interestingly, while fucosylation in IgG1 is positively associated with fucosylation in IgG2/3, fucosylation shows relatively weak or no consistent correlations (positive or negative) with most other derived glycosylation traits, such as bisection, suggesting that fucosylation may vary more independently from the galactosylation–sialylation–bisection axis.

LMMs were used to assess batch and cohort effects on IgG *N*-glycan traits, adjusting for age, sex, smoking, alcohol consumption, and BMI. Despite most models showing statistically significant likelihood ratio tests (LRT) for both batch and cohort effects (p < 0·05), the intraclass correlation coefficients (ICC) indicated minimal practical impact. Batch effects accounted for just 1.5-1.8% of the total variance, while cohort effects contributed only 0·3-0·4%, reflecting a very small proportion of overall variability. Given the low ICC values, both batch and cohort effects were excluded from the final model to simplify interpretation without significantly reducing model fit.

The effects of including BMI in the regression models were evaluated by comparing Model 2 (without BMI) and Model 3 (with BMI). Including BMI generally improved model fit for most glycan traits (P < 0·05). Changes in coefficients were mostly minor (< 0·005), suggesting a minimal impact of BMI as mediator between other predictors and glycan outcomes. To further explore the findings related to BMI and evaluate the impact of central adiposity, waist circumference was included in Model 3 as a replacement for BMI, and findings were consistent with BMI across all outcomes.

In this study, 95·8% of participants were of European, Middle Eastern, or North African descent and 4·2% belonged to other ethnic backgrounds. IgG subclass distributions were 70% versus 69% for IgG1, 26% versus 24% for IgG2/3, and 4% versus 7% for IgG4. Stratified analysis showed no notable differences in effect size or direction across determinants, albeit loss of significant results in the smaller ‘other ethnicity’ subgroup. Including ASCCEG classification or binary ancestry variable in the models did not alter the results, indicating ethnicity did not introduce confounding bias.

Finally, an exploratory analysis stratified by menopausal status (450 pre-/perimenopausal and 1,375 postmenopausal women) demonstrated largely consistent directions of association across strata, albeit with differences in effect magnitude. This resulted in comparable overall glycosylation patterns and concordance with the main cross-sectional analyses for all determinants (Supplementary Figure S7).

# **3. Supplementary Tables**

# **Supplementary Table S1 Glycan compositions of the IgG *N*-glycome**

The table includes glycan composition, structure, short name, Oxford notation, GlyTouCan identities (International Glycan Structure Repository), UPLC glycan peak assignments, and presumed functions. Derived traits—core fucosylation, bisecting N-acetylglucosamine (bisection), galactosylation, sialylation, and sialylation per galactose—were calculated by summing relative areas of structurally similar glycans. Fucosylation in IgG4 could not be reliably measured due to the relatively low abundance of IgG4 and the interference of IgG1 fucosylated glycoforms with IgG4 afucosylated glycoforms.

| **Glycan composition** | **Structure** | **Glycan short name** | **Oxford notation name*** | **GlyTouCan composition/structure^#^** | **UPLC glycan peak**** | **Presumed function** |
| --- | --- | --- | --- | --- | --- | --- |
| H3N3F1 |  | G0F-N | F(6)A1 | G94854LT / G07483YN | GP1 |  |
| H3N4 |  | G0 | A2 | na / G39213VZ | GP2 |  |
| H3N4F1 |  | G0F | F(6)A2 | G49874UX / G80858MF | GP4 |  |
| H3N5 |  | G0N | A2B | G31936TA / G59471TH | GP3 |  |
| H3N5F1 |  | G0NF | F(6)A2B | G10256JP / G30159WR | GP6 (GP7) |  |
| H4N4 |  | G1 | A2G1 | G81295CK / G63640QH | GP7 (GP6) |  |
| H4N4F1 |  | G1F | F(6)A2G1 | G46687AB / na | GP9 (GP8a/b) |  |
| H4N4F1S1 |  | G1S1F | F(6)A2G1S1 | G14994KB / na | GP16b (GP16a) |  |
| H4N5 |  | G1N | A2BG1 | G23432EQ / na | GP8a |  |
| H4N5F1 |  | G1FN | F(6)A2BG1 | G03382KH / na | GP10/11 (GP12) |  |
| H4N5F1S1 |  | G1S1FN | F(6)A2BG1S1 | G72667IM / na | GP17 (GP16b)*** |  |
| H5N4 |  | G2 | A2G2 | G50045TK / G36191CD | GP12 |  |
| H5N4F1 |  | G2F | F(6)A2G2 | G23863VK / G78059CC | GP14 (GP13,GP15) |  |
| H5N4F1S1 |  | G2S1F | F(6)A2G2S1 | G84452RH / G81413UE | GP18b (GP18a) |  |
| H5N4F1S2 |  | G2S2F | F(6)A2G2S2 | G22310AV / G56749GV | GP23 |  |
| H5N4S1 |  | G2S1 | A2G2S1 | G06356OH / na | GP17 |  |
| H5N5 |  | G2N | A2BG2 | G79568CQ / G52934AK | GP13 |  |
| H5N5F1 |  | G2FN | F(6)A2BG2 | G91636VS / G25520XG | GP15 |  |
| H5N5F1S1 |  | G2S1FN | F(6)A2BG2S1 | G02030ZB / G86632ZU | GP19 |  |
| Fucosylation |  | F | F |  |  | **Anti-inflammatory** |
| Bisection |  | N | B |  |  | **Pro-inflammatory** |
| Galactosylation |  | G | G |  |  | **Anti-inflammatory** |
| Sialylation |  | S | S |  |  | **Anti-inflammatory** |
| Sialylation per galactose |  | S/G | S/G |  |  | **Anti-inflammatory** |

*Connotation of linkages removed as MS does not specify linkage type.

**UPLC glycan peak that most closely resembles the glycan composition (i.e., the composition that entails the highest percentage of the glycan peak). Peaks in parentheses also (partly) contain the composition.

***11% of GP17, 9% of GP16.

^#^ <https://glytoucan.org/> ; structure and linkages are assigned based on literature consensus. See, for example DOI:10.1002/pmic.200700968; na = desired composition/structure not available in GlyTouCan

**Supplementary Table S2 Baseline Characteristics of Participants with Repeated IgG N-Glycosylation Measurements**

This table presents the baseline characteristics of the 448 participants with repeated IgG glycosylation measurements, including age, body mass index (BMI), smoking status, alcohol consumption, and follow-up time, stratified by sex. These participants are included in the longitudinal linear mixed model (LMM) analysis.

|  | **Male (N=188)** | **Female (N=260)** | **Overall (N=448)** |
| --- | --- | --- | --- |
| **Age (years)** |  |  |  |
| Mean (SD) | 57·1 (5·66) | 57·0 (5·71) | 57·0 (5·68) |
| Median [Min, Max] | 57·2 [46·8, 78·2] | 57·1 [46·6, 84·2] | 57·1 [46·6, 84·2] |
| **Body Mass Index (kg/m²)** |  |  |  |
| Mean (SD) | 27·5 (3·83) | 27·2 (4·92) | 27·3 (4·49) |
| Median [Min, Max] | 26·7 [20·0, 42·6] | 26·2 [17·1, 50·2] | 26·6 [17·1, 50·2] |
| **Smoking Status** |  |  |  |
| Never | 65 (34·6%) | 86 (33·1%) | 151 (33·7%) |
| Former | 90 (47·9%) | 129 (49·6%) | 219 (48·9%) |
| Current | 33 (17·6%) | 45 (17·3%) | 78 (17·4%) |
| **Alcohol Consumption** |  |  |  |
| Mild | 124 (66·0%) | 219 (84·2%) | 343 (76·6%) |
| Moderate | 43 (22·9%) | 40 (15·4%) | 83 (18·5%) |
| Heavy | 21 (11·2%) | 1 (0·4%) | 22 (4·9%) |
| **Follow-up Time (years)** |  |  |  |
| Mean (SD) | 5·64 (0·468) | 5·61 (0·429) | 5·62 (0·445) |
| Median [Min, Max] | 5·54 [4·48, 7·67] | 5·52 [4·84, 7·92] | 5·52 [4·48, 7·92] |

**Supplementary Table S3.1 Effects of demographic and lifestyle determinants on the main IgG *N*-glycan derived traits**
Table S3.1 presents the cross-sectional linear regression results evaluating the effects of demographic and lifestyle determinants and the four main derived traits. Estimates represent the change in *N*-glycan derived traits in standard deviations (SD). Estimates are expressed relative to male sex, never smoking, and mild alcohol consumption, respectively. Models were adjusted for age, sex, smoking, alcohol consumption, and BMI. Statistically significant effects are displayed in bold (P<0.05). Derived traits generally considered anti‑inflammatory are shown in green and pro‑inflammatory traits in red.

|  | Age (per 1 unit increase) | | Female sex | | BMI (per 1 unit increase) | | Current smoking | | Heavy alcohol consumption | |
| --- | --- | --- | --- | --- | --- | --- | --- | --- | --- | --- |
|  | Estimate (95% CI) | P-value | Estimate (95% CI) | P-value | Estimate (95% CI) | P-value | Estimate (95% CI) | P-value | Estimate (95% CI) | P-value |
| Fucosylation |  |  |  |  |  |  |  |  |  |  |
| IgG1 | **0·01 (0·01, 0·01)** | **<0·001** | -0·03 (-0·08, 0·01) | 0·152 | **0·02 (0·01, 0·02)** | **<0·001** | **-0·18 (-0·25, -0·12)** | **<0·001** | -0·01 (-0·08, 0·05) | 0·702 |
| IgG2/3 | **0·01 (0·00, 0·01)** | **<0·001** | **-0·11 (-0·16, -0·06)** | **<0·001** | **0·01 (0·00, 0·01)** | **0·010** | **-0·32 (-0·38, -0·25)** | **<0·001** | **-0·13 (-0·20, -0·05)** | **0·001** |
| Bisection |  |  |  |  |  |  |  |  |  |  |
| IgG1 | **0·03 (0·03, 0·03)** | **<0·001** | **0·10 (0·06, 0·15)** | **<0·001** | **0·02 (0·01, 0·02)** | **<0·001** | **0·62 (0·56, 0·68)** | **<0·001** | **0·11 (0·04, 0·18)** | **0·001** |
| IgG2/3 | **0·03 (0·02, 0·03)** | **<0·001** | **0·21 (0·17, 0·26)** | **<0·001** | 0·00 (-0·00, 0·01) | 0·064 | **0·82 (0·76, 0·88)** | **<0·001** | **0·10 (0·03, 0·17)** | **0·005** |
| IgG4 | **0·03 (0·02, 0·03)** | **<0·001** | **0·33 (0·28, 0·38)** | **<0·001** | **0·02 (0·01, 0·02)** | **<0·001** | **0·66 (0·59, 0·73)** | **<0·001** | **0·10 (0·03, 0·18)** | **0·007** |
| Galactosylation |  |  |  |  |  |  |  |  |  |  |
| IgG1 | **-0·06 (-0·06, -0·05)** | **<0·001** | **-0·04 (-0·08, -0·00)** | **0·033** | **-0·03 (-0·04, -0·03)** | **<0·001** | **-0·09 (-0·14, -0·03)** | **0·001** | -0·02 (-0·08, 0·03) | 0·391 |
| IgG2/3 | **-0·06 (-0·06, -0·06)** | **<0·001** | **-0·11 (-0·15, -0·07)** | **<0·001** | **-0·01 (-0·02, -0·01)** | **<0·001** | **0·06 (0·01, 0·11)** | **0·023** | 0·02 (-0·04, 0·07) | 0·589 |
| IgG4 | **-0·05 (-0·05, -0·04)** | **<0·001** | **0·07 (0·02, 0·12)** | **0·005** | **-0·04 (-0·04, -0·03)** | **<0·001** | **-0·07 (-0·13, -0·00)** | **0·044** | -0·04 (-0·11, 0·04) | 0·334 |
| Sialylation |  |  |  |  |  |  |  |  |  |  |
| IgG1 | **-0·05 (-0·05, -0·05)** | **<0·001** | **-0·07 (-0·11, -0·03)** | **0·001** | **-0·02 (-0·03, -0·02)** | **<0·001** | **-0·08 (-0·13, -0·02)** | **0·006** | -0·02 (-0·08, 0·04) | 0·545 |
| IgG2/3 | **-0·04 (-0·05, -0·04)** | **<0·001** | **-0·14 (-0·18, -0·09)** | **<0·001** | **-0·01 (-0·01, -0·00)** | **0·003** | **0·08 (0·03, 0·14)** | **0·005** | **0·11 (0·04, 0·18)** | **0·002** |
| IgG4 | **-0·04 (-0·04, -0·04)** | **<0·001** | **0·06 (0·01, 0·11)** | **0·015** | **-0·03 (-0·04, -0·03)** | **<0·001** | **-0·10 (-0·17, -0·03)** | **0·004** | -0·03 (-0·10, 0·05) | 0·513 |

**Supplementary Table S4 Effect of binary time on IgG N-glycosylation traits estimated using linear mixed models**
Each row represents the fixed effect estimate for the time variable (follow-up vs. baseline) in relation to a specific IgG glycosylation outcome. Models were adjusted for age, sex, smoking, alcohol consumption, and BMI, with a random intercept for participant ID. False discovery rate (FDR)–adjusted p-values were calculated per IgG subclass group, with no correction for IgG derived traits. Significance is indicated as follows: p < 0·05 (*), p < 0·01 (**), p < 0·001 (***).

| **Outcome** | **Estimate** | **Std·Error** | **p-value** | **FDR-adjusted** | **Significance** |
| --- | --- | --- | --- | --- | --- |
| IgGI_fucosylation | -0·007 | 0·019 | 7·19e-01 | 7·19e-01 |  |
| IgGI_bisection | 0·279 | 0·018 | 0·00e+00 | 0·00e+00 | *** |
| IgGI_galactosylation | -0·369 | 0·027 | 0·00e+00 | 0·00e+00 | *** |
| IgGI_sialylation | -0·313 | 0·029 | 0·00e+00 | 0·00e+00 | *** |
| IgGI_sialylation_per_galactose | -0·301 | 0·028 | 0·00e+00 | 0·00e+00 | *** |
| IgGII_fucosylation | -0·457 | 0·025 | 0·00e+00 | 0·00e+00 | *** |
| IgGII_bisection | 0·239 | 0·018 | 0·00e+00 | 0·00e+00 | *** |
| IgGII_galactosylation | -0·307 | 0·025 | 0·00e+00 | 0·00e+00 | *** |
| IgGII_sialylation | -0·338 | 0·029 | 0·00e+00 | 0·00e+00 | *** |
| IgGII_sialylation_per_galactose | -0·298 | 0·028 | 0·00e+00 | 0·00e+00 | *** |
| IgGIV_bisection | 0·165 | 0·023 | 0·00e+00 | 0·00e+00 | *** |
| IgGIV_galactosylation | -0·393 | 0·027 | 0·00e+00 | 0·00e+00 | *** |
| IgGIV_sialylation | -0·490 | 0·030 | 0·00e+00 | 0·00e+00 | *** |
| IgGIV_sialylation_per_galactose | -0·500 | 0·030 | 0·00e+00 | 0·00e+00 | *** |
| IgGI1H3N3F1 | -0·249 | 0·047 | 2·00e-07 | 2·00e-07 | *** |
| IgGI1H3N4 | 0·300 | 0·042 | 0·00e+00 | 0·00e+00 | *** |
| IgGI1H3N4F1 | 0·268 | 0·024 | 0·00e+00 | 0·00e+00 | *** |
| IgGI1H3N5 | -0·186 | 0·031 | 0·00e+00 | 0·00e+00 | *** |
| IgGI1H3N5F1 | 0·486 | 0·024 | 0·00e+00 | 0·00e+00 | *** |
| IgGI1H4N4 | 0·088 | 0·016 | 1·00e-07 | 1·00e-07 | *** |
| IgGI1H4N4F1 | -0·288 | 0·026 | 0·00e+00 | 0·00e+00 | *** |
| IgGI1H4N4F1S1 | 0·066 | 0·029 | 2·21e-02 | 2·21e-02 | * |
| IgGI1H4N5 | -0·285 | 0·022 | 0·00e+00 | 0·00e+00 | *** |
| IgGI1H4N5F1 | 0·170 | 0·017 | 0·00e+00 | 0·00e+00 | *** |
| IgGI1H4N5F1S1 | -0·126 | 0·022 | 0·00e+00 | 0·00e+00 | *** |
| IgGI1H5N4 | -0·167 | 0·025 | 0·00e+00 | 0·00e+00 | *** |
| IgGI1H5N4F1 | -0·322 | 0·027 | 0·00e+00 | 0·00e+00 | *** |
| IgGI1H5N4F1S1 | -0·245 | 0·029 | 0·00e+00 | 0·00e+00 | *** |
| IgGI1H5N4F1S2 | -0·578 | 0·038 | 0·00e+00 | 0·00e+00 | *** |
| IgGI1H5N4S1 | -0·396 | 0·027 | 0·00e+00 | 0·00e+00 | *** |
| IgGI1H5N5 | -0·480 | 0·042 | 0·00e+00 | 0·00e+00 | *** |
| IgGI1H5N5F1 | -0·150 | 0·023 | 0·00e+00 | 0·00e+00 | *** |
| IgGI1H5N5F1S1 | -0·464 | 0·029 | 0·00e+00 | 0·00e+00 | *** |
| IgGII1H3N4 | 0·500 | 0·024 | 0·00e+00 | 0·00e+00 | *** |
| IgGII1H3N4F1 | 0·229 | 0·022 | 0·00e+00 | 0·00e+00 | *** |
| IgGII1H3N5F1 | 0·372 | 0·023 | 0·00e+00 | 0·00e+00 | *** |
| IgGII1H4N4 | 0·417 | 0·025 | 0·00e+00 | 0·00e+00 | *** |
| IgGII1H4N4F1 | -0·422 | 0·023 | 0·00e+00 | 0·00e+00 | *** |
| IgGII1H4N4F1S1 | -0·111 | 0·029 | 1·70e-04 | 1·87e-04 | *** |
| IgGII1H4N5F1 | 0·027 | 0·016 | 8·79e-02 | 8·79e-02 |  |
| IgGII1H5N4 | 0·340 | 0·028 | 0·00e+00 | 0·00e+00 | *** |
| IgGII1H5N4F1 | -0·204 | 0·027 | 0·00e+00 | 0·00e+00 | *** |
| IgGII1H5N4F1S1 | -0·387 | 0·030 | 0·00e+00 | 0·00e+00 | *** |
| IgGII1H5N5F1 | -0·173 | 0·023 | 0·00e+00 | 0·00e+00 | *** |
| IgGIV1H3N4F1 | 0·358 | 0·026 | 0·00e+00 | 0·00e+00 | *** |
| IgGIV1H3N5F1 | 0·264 | 0·025 | 0·00e+00 | 0·00e+00 | *** |
| IgGIV1H4N4F1 | 0·002 | 0·026 | 9·48e-01 | 9·48e-01 |  |
| IgGIV1H4N4F1S1 | -0·321 | 0·029 | 0·00e+00 | 0·00e+00 | *** |
| IgGIV1H4N5F1 | -0·088 | 0·023 | 1·23e-04 | 1·44e-04 | *** |
| IgGIV1H5N4F1 | -0·285 | 0·026 | 0·00e+00 | 0·00e+00 | *** |
| IgGIV1H5N4F1S1 | -0·459 | 0·030 | 0·00e+00 | 0·00e+00 | *** |

**Supplementary Table S5 Effect of continuous time (years since baseline) on IgG N-glycosylation traits estimated using linear mixed models**
Each row represents the estimated annual change in a glycosylation trait. Models were adjusted for age, sex, smoking, alcohol consumption, and BMI, with a random intercept for participant ID. False discovery rate (FDR)–adjusted p-values were calculated per IgG subclass group, with no correction for IgG derived traits. Significance is indicated as follows: p < 0·05 (*), p < 0·01 (**), p < 0·001 (***).

| **Outcome** | **Estimate** | **Std·Error** | **p-value** | **FDR-adjusted** | **Significance** |
| --- | --- | --- | --- | --- | --- |
| IgGI_fucosylation | -0·001 | 0·003 | 6·99e-01 | 6·99e-01 |  |
| IgGI_bisection | 0·050 | 0·003 | 0·00e+00 | 0·00e+00 | *** |
| IgGI_galactosylation | -0·066 | 0·005 | 0·00e+00 | 0·00e+00 | *** |
| IgGI_sialylation | -0·056 | 0·005 | 0·00e+00 | 0·00e+00 | *** |
| IgGI_sialylation_per_galactose | -0·054 | 0·005 | 0·00e+00 | 0·00e+00 | *** |
| IgGII_fucosylation | -0·081 | 0·004 | 0·00e+00 | 0·00e+00 | *** |
| IgGII_bisection | 0·043 | 0·003 | 0·00e+00 | 0·00e+00 | *** |
| IgGII_galactosylation | -0·055 | 0·004 | 0·00e+00 | 0·00e+00 | *** |
| IgGII_sialylation | -0·061 | 0·005 | 0·00e+00 | 0·00e+00 | *** |
| IgGII_sialylation_per_galactose | -0·053 | 0·005 | 0·00e+00 | 0·00e+00 | *** |
| IgGIV_bisection | 0·030 | 0·004 | 0·00e+00 | 0·00e+00 | *** |
| IgGIV_galactosylation | -0·070 | 0·005 | 0·00e+00 | 0·00e+00 | *** |
| IgGIV_sialylation | -0·087 | 0·005 | 0·00e+00 | 0·00e+00 | *** |
| IgGIV_sialylation_per_galactose | -0·089 | 0·005 | 0·00e+00 | 0·00e+00 | *** |
| IgGI1H3N3F1 | -0·040 | 0·008 | 3·40e-06 | 3·60e-06 | *** |
| IgGI1H3N4 | 0·053 | 0·007 | 0·00e+00 | 0·00e+00 | *** |
| IgGI1H3N4F1 | 0·048 | 0·004 | 0·00e+00 | 0·00e+00 | *** |
| IgGI1H3N5 | -0·032 | 0·005 | 0·00e+00 | 0·00e+00 | *** |
| IgGI1H3N5F1 | 0·087 | 0·004 | 0·00e+00 | 0·00e+00 | *** |
| IgGI1H4N4 | 0·016 | 0·003 | 0·00e+00 | 0·00e+00 | *** |
| IgGI1H4N4F1 | -0·051 | 0·005 | 0·00e+00 | 0·00e+00 | *** |
| IgGI1H4N4F1S1 | 0·012 | 0·005 | 1·70e-02 | 1·70e-02 | * |
| IgGI1H4N5 | -0·050 | 0·004 | 0·00e+00 | 0·00e+00 | *** |
| IgGI1H4N5F1 | 0·030 | 0·003 | 0·00e+00 | 0·00e+00 | *** |
| IgGI1H4N5F1S1 | -0·022 | 0·004 | 0·00e+00 | 0·00e+00 | *** |
| IgGI1H5N4 | -0·030 | 0·004 | 0·00e+00 | 0·00e+00 | *** |
| IgGI1H5N4F1 | -0·058 | 0·005 | 0·00e+00 | 0·00e+00 | *** |
| IgGI1H5N4F1S1 | -0·044 | 0·005 | 0·00e+00 | 0·00e+00 | *** |
| IgGI1H5N4F1S2 | -0·103 | 0·007 | 0·00e+00 | 0·00e+00 | *** |
| IgGI1H5N4S1 | -0·071 | 0·005 | 0·00e+00 | 0·00e+00 | *** |
| IgGI1H5N5 | -0·085 | 0·007 | 0·00e+00 | 0·00e+00 | *** |
| IgGI1H5N5F1 | -0·027 | 0·004 | 0·00e+00 | 0·00e+00 | *** |
| IgGI1H5N5F1S1 | -0·082 | 0·005 | 0·00e+00 | 0·00e+00 | *** |
| IgGII1H3N4 | 0·089 | 0·004 | 0·00e+00 | 0·00e+00 | *** |
| IgGII1H3N4F1 | 0·041 | 0·004 | 0·00e+00 | 0·00e+00 | *** |
| IgGII1H3N5F1 | 0·067 | 0·004 | 0·00e+00 | 0·00e+00 | *** |
| IgGII1H4N4 | 0·074 | 0·004 | 0·00e+00 | 0·00e+00 | *** |
| IgGII1H4N4F1 | -0·074 | 0·004 | 0·00e+00 | 0·00e+00 | *** |
| IgGII1H4N4F1S1 | -0·020 | 0·005 | 1·54e-04 | 1·69e-04 | *** |
| IgGII1H4N5F1 | 0·005 | 0·003 | 6·87e-02 | 6·87e-02 |  |
| IgGII1H5N4 | 0·060 | 0·005 | 0·00e+00 | 0·00e+00 | *** |
| IgGII1H5N4F1 | -0·037 | 0·005 | 0·00e+00 | 0·00e+00 | *** |
| IgGII1H5N4F1S1 | -0·070 | 0·005 | 0·00e+00 | 0·00e+00 | *** |
| IgGII1H5N5F1 | -0·031 | 0·004 | 0·00e+00 | 0·00e+00 | *** |
| IgGIV1H3N4F1 | 0·064 | 0·005 | 0·00e+00 | 0·00e+00 | *** |
| IgGIV1H3N5F1 | 0·047 | 0·004 | 0·00e+00 | 0·00e+00 | *** |
| IgGIV1H4N4F1 | 0·000 | 0·005 | 9·76e-01 | 9·76e-01 |  |
| IgGIV1H4N4F1S1 | -0·058 | 0·005 | 0·00e+00 | 0·00e+00 | *** |
| IgGIV1H4N5F1 | -0·015 | 0·004 | 2·21e-04 | 2·58e-04 | *** |
| IgGIV1H5N4F1 | -0·051 | 0·005 | 0·00e+00 | 0·00e+00 | *** |
| IgGIV1H5N4F1S1 | -0·082 | 0·005 | 0·00e+00 | 0·00e+00 | *** |

**Supplementary Table S6 Adjusted and unadjusted Intraclass Correlation Coefficients**
Each row represents the unadjusted and time-adjusted Intraclass Correlation Coefficients (ICCs) for each glycosylation trait. ICCs were estimated using linear mixed-effects models, with time included as a fixed effect in the adjusted models. Higher ICC values indicate greater stability of the trait across measurements, while lower values reflect higher within-individual variability.

| **Outcome** | **ICC unadjusted** | **ICC adjusted** |
| --- | --- | --- |
| IgGI_fucosylation | 0·92 | 0·92 |
| IgGI_bisection | 0·91 | 0·93 |
| IgGI_galactosylation | 0·81 | 0·84 |
| IgGI_sialylation | 0·79 | 0·81 |
| IgGI_sialylation_per_galactose | 0·80 | 0·82 |
| IgGII_fucosylation | 0·81 | 0·86 |
| IgGII_bisection | 0·91 | 0·93 |
| IgGII_galactosylation | 0·84 | 0·86 |
| IgGII_sialylation | 0·79 | 0·81 |
| IgGII_sialylation_per_galactose | 0·80 | 0·82 |
| IgGIV_bisection | 0·90 | 0·91 |
| IgGIV_galactosylation | 0·84 | 0·87 |
| IgGIV_sialylation | 0·79 | 0·84 |
| IgGIV_sialylation_per_galactose | 0·79 | 0·84 |
| IgGI1H3N3F1 | 0·48 | 0·49 |
| IgGI1H3N4 | 0·59 | 0·60 |
| IgGI1H3N4F1 | 0·86 | 0·87 |
| IgGI1H3N5 | 0·78 | 0·79 |
| IgGI1H3N5F1 | 0·81 | 0·86 |
| IgGI1H4N4 | 0·94 | 0·94 |
| IgGI1H4N4F1 | 0·83 | 0·84 |
| IgGI1H4N4F1S1 | 0·82 | 0·82 |
| IgGI1H4N5 | 0·87 | 0·89 |
| IgGI1H4N5F1 | 0·93 | 0·93 |
| IgGI1H4N5F1S1 | 0·88 | 0·89 |
| IgGI1H5N4 | 0·86 | 0·86 |
| IgGI1H5N4F1 | 0·81 | 0·84 |
| IgGI1H5N4F1S1 | 0·80 | 0·81 |
| IgGI1H5N4F1S2 | 0·59 | 0·65 |
| IgGI1H5N4S1 | 0·80 | 0·84 |
| IgGI1H5N5 | 0·55 | 0·58 |
| IgGI1H5N5F1 | 0·88 | 0·89 |
| IgGI1H5N5F1S1 | 0·76 | 0·80 |
| IgGII1H3N4 | 0·81 | 0·86 |
| IgGII1H3N4F1 | 0·88 | 0·89 |
| IgGII1H3N5F1 | 0·85 | 0·88 |
| IgGII1H4N4 | 0·82 | 0·86 |
| IgGII1H4N4F1 | 0·83 | 0·87 |
| IgGII1H4N4F1S1 | 0·81 | 0·81 |
| IgGII1H4N5F1 | 0·94 | 0·94 |
| IgGII1H5N4 | 0·80 | 0·82 |
| IgGII1H5N4F1 | 0·83 | 0·84 |
| IgGII1H5N4F1S1 | 0·76 | 0·79 |
| IgGII1H5N5F1 | 0·88 | 0·89 |
| IgGIV1H3N4F1 | 0·85 | 0·88 |
| IgGIV1H3N5F1 | 0·87 | 0·89 |
| IgGIV1H4N4F1 | 0·88 | 0·88 |
| IgGIV1H4N4F1S1 | 0·83 | 0·85 |
| IgGIV1H4N5F1 | 0·91 | 0·91 |
| IgGIV1H5N4F1 | 0·87 | 0·88 |
| IgGIV1H5N4F1S1 | 0·79 | 0·84 |

# **4. Supplementary Figures**

**Supplementary Figure S1 Directed acyclic graph (DAG) illustrating hypothesized causal relationships among independent and dependent variables**

Independent variables include modifiable (body mass index [BMI], waist-to-hip ratio [WHR], smoking, alcohol) and non-modifiable factors (sex and age), with the dependent variable IgG N-glycosylation. Unmeasured baseline variables (Smoking T0 and Alcohol T0) are included to capture prior exposures that influence measured smoking (Smoking T1) and alcohol (Alcohol T1) levels. The DAG reflects the complex interactions among these factors.

**Supplementary Fig. S2 Distribution of IgG Glycan Traits Across Subclasses**

This figure shows violin plots for various IgG glycan traits across subclasses 1-4, including **fucosylation, bisection**, **galactosylation, sialylation**, and **sialylation per galactose**. The y-axis represents the percentage of the total glycan signal for each trait, reflecting their relative abundance. The width of each violin indicates the density of the data, capturing the distribution of each glycan trait. Colors are used for visual differentiation only and do not indicate any specific order or magnitude. Notably, fucosylation and sialylation traits show concentrated distributions, while traits like galactosylation display broader variability, indicating greater heterogeneity in these subclasses.

**A**

**B**

**C**

**Supplemental Fig. S3 Assay performance**

Relative abundance of A) glycosylation traits, B) major glycans, and C) minor glycans are displayed per IgG subclass. Bar graphs and whiskers show the mean and standard deviation of 392, 388, and 379 replicate measurements of the Visucon standard, equally distributed over all batches. Red and black dots indicate the individual measurements of the standard and the samples, respectively. This overview underlines the high precision of the utilized LC-MS assay. Furthermore, it shows that relevant clinical differences are expected to be accessible since the biological variation measured within the cohort is several magnitudes larger than the technical variability.

**Supplementary Fig. S4 Correlation matrix showing the relationships between derived glycosylation traits across IgG subclasses 1, 2/3, and 4.** The matrix displays Pearson correlation coefficients for each trait pair, with significance stars indicating the level of statistical significance (** p < 0·001, ** p < 0·01, * p < 0·05). The color gradient ranges from blue (negative correlation) to red (positive correlation), with white indicating no correlation.

**Supplementary Fig. S5 Flow chart outlines the inclusion and exclusion of participants.**

This flow chart outlines the participant inclusion and exclusion for the IgG glycosylation analysis. Of the initial 8,911 samples, 176 were excluded due to undetectable glycosylation measurements. Of these, 499 were excluded due to repeated measurements, resulting in the final sample of 8,242 participants. Further exclusion is based on specific subclass missing data, with final cohort sizes of 8,210 for IgG1, 8,023 for IgG2/3, and 6130 for IgG4.

**Supplementary Fig. S6·1 Age-Sex Interaction Effects on IgG Glycan Traits in Individuals Aged 45 - 55**

**-- Page 1 of 4**

This figure presents the interaction effects between **age** and **sex** on various **IgG glycan traits** in individuals aged **45-55, adjusted for smoking, alcohol, and body mass index.** The first page shows the **glycan traits** ordered by significance, while the following pages present the **individual glycan compositions** in order of significance. Separate regression lines are shown for **males (blue)** and **females (red),** with shaded areas representing **95% confidence intervals**. The p-values for the overall interaction term (age × sex), corrected using the false discovery rate (FDR), are displayed directly on each plot. The y-axis is standardized (-1.5 to 2) to enable comparison.

**Supplementary Fig. S6·2 Age-Sex Interaction Effects on IgG Glycan Compositions in Individuals Aged 45 - 55 -- Page 2 of 4**

**Supplementary Fig. S6·3 Age-Sex Interaction Effects on IgG Glycan Compositions in Individuals Aged 45 - 55 -- Page 3 of 4**

**Supplementary Fig. S6·4 Age-Sex Interaction Effects on IgG Glycan Compositions in Individuals Aged 45 - 55 -- Page 4 of 4**

**Supplementary Fig. S7·1 Analyses Stratified by Menopause Status**

The figure presents the estimated effects of age (A and B) and BMI (C and D) on IgG *N*-glycosylation traits and compositions in women of RSIII, stratified based on menopause status, with β estimates on the y-axis and individual traits on the x-axis. A vertical dashed line separates derived traits from individual glycan compositions. Data points are color-coded by IgG subclass, with green representing IgG1, orange representing IgG2/3, and purple representing IgG4. Grey circles indicate traits that did not pass the significance threshold (P < 0**·**05). Traits are additionally color-coded by inflammatory status, with green indicating anti-inflammatory traits and red indicating pro-inflammatory traits. All estimates are adjusted for age, smoking, alcohol consumption, and BMI.

**Supplementary Fig. S7·2 Analyses Stratified by Menopause Status**

The figure presents the estimated effects of smoking (A and B) and alcohol consumption (C and D) on IgG N-glycosylation traits and compositions in women of RSIII, stratified based on menopause status, with β estimates on the y-axis and individual traits on the x-axis. A vertical dashed line separates derived traits from individual glycan compositions. Data points are distinguished by shape, with circles representing IgG1, triangles representing IgG2/3, and diamonds representing IgG4. All estimates are adjusted for age, smoking, alcohol consumption, and BMI.

**Supplementary Fig. S8·1 Longitudinal Changes in IgG Glycans Over Time – Page 1 of 4**

Results from linear mixed models (LMM) assessing changes in IgG glycan traits over an average follow-up period of 5.6 years (binary variable of time), adjusting for age, sex, BMI, smoking, and alcohol consumption, with a random effect for individual ID to account for within-subject variation. Y-axis shows modeled Z-standardized glycan values estimated from the fixed effects of our LMM, representing average population-level estimates, assuming mean age, male sex, mean BMI, non-smoker status, and mild alcohol consumption. The first page presents derived glycan traits for each IgG subclass (IgG1, IgG2/3, IgG4), ordered by significance, while pages 2-4 display individual glycan compositions, also ranked by significance. All p-values of the individual compositions are FDR-corrected to account for multiple testing and significant results are indicated with **asterisks**.

**Supplementary Fig. S8·2 Longitudinal Changes in IgG Glycans Over Time — Page 2 of 4**

**Supplementary Fig. S8·3 Longitudinal Changes in IgG Glycans Over Time — Page 3 of 4**

**Supplementary Fig. S8·4 Longitudinal Changes in IgG Glycans Over Time—Page 4 of 4**

# **5. Supplementary References**

1.	Falck D, Wuhrer M: **GlYcoLISA: antigen-specific and subclass-specific IgG Fc glycosylation analysis based on an immunosorbent assay with an LC-MS readout**. *Nat Protoc* 2024, **19**(6):1887-1909.

2.	Zauner G, Selman MH, Bondt A, Rombouts Y, Blank D, Deelder AM, Wuhrer M: **Glycoproteomic analysis of antibodies**. *Mol Cell Proteomics* 2013, **12**(4):856-865.

3.	Krištić J, Lauc G: **The importance of IgG glycosylation-What did we learn after analyzing over 100,000 individuals**. *Immunol Rev* 2024, **328**(1):143-170.

4.	Shkunnikova S, Mijakovac A, Sironic L, Hanic M, Lauc G, Kavur MM: **IgG glycans in health and disease: Prediction, intervention, prognosis, and therapy**. *Biotechnol Adv* 2023, **67**:108169.

5.	Cobb BA: **The history of IgG glycosylation and where we are now**. *Glycobiology* 2020, **30**(4):202-213.

6.	Gudelj I, Lauc G, Pezer M: **Immunoglobulin G glycosylation in aging and diseases**. *Cell Immunol* 2018, **333**:65-79.

7.	Dekkers G, Treffers L, Plomp R, Bentlage AEH, de Boer M, Koeleman CAM, Lissenberg-Thunnissen SN, Visser R, Brouwer M, Mok JY *et al*: **Decoding the Human Immunoglobulin G-Glycan Repertoire Reveals a Spectrum of Fc-Receptor- and Complement-Mediated-Effector Activities**. *Frontiers in Immunology* 2017, **8**.

8.	Pereira NA, Chan KF, Lin PC, Song Z: **The "less-is-more" in therapeutic antibodies: Afucosylated anti-cancer antibodies with enhanced antibody-dependent cellular cytotoxicity**. *MAbs* 2018, **10**(5):693-711.

9.	Mimura Y, Mimura-Kimura Y, Saldova R, Rudd PM, Jefferis R: **Enhanced Immunomodulatory Effect of Intravenous Immunoglobulin by Fc Galactosylation and Nonfucosylation**. *Front Immunol* 2022, **13**:818382.

10.	Washburn N, Schwab I, Ortiz D, Bhatnagar N, Lansing JC, Medeiros A, Tyler S, Mekala D, Cochran E, Sarvaiya H *et al*: **Controlled tetra-Fc sialylation of IVIg results in a drug candidate with consistent enhanced anti-inflammatory activity**. *Proc Natl Acad Sci U S A* 2015, **112**(11):E1297-1306.

11.	Rademacher TW, Williams P, Dwek RA: **Agalactosyl glycoforms of IgG autoantibodies are pathogenic**. *Proc Natl Acad Sci U S A* 1994, **91**(13):6123-6127.

12.	Ohmi Y, Ise W, Harazono A, Takakura D, Fukuyama H, Baba Y, Narazaki M, Shoda H, Takahashi N, Ohkawa Y *et al*: **Sialylation converts arthritogenic IgG into inhibitors of collagen-induced arthritis**. *Nat Commun* 2016, **7**:11205.

13.	Burls A: **What is critical appraisal?** In*.*: Hayward Medical Communications; 2014.
